# Gastrotomy for Hæmatemesis

**Published:** 1900-10-27

**Authors:** 


### GASTROTOMY FOR HÆMATEMESIS.

At the same meeting Mr. Mansell Moullin read a paper on three cases in which he had performed gastrotomy for the treatment of hæmatemesis.

In the first case, on the stomach being opened and carefully examined, two small superficial ulcers were found on the posterior wall, nearer the cardiac than the pyloric end. Silk ligatures were passed in two directions under the ulcers and tied so as to strangulate the base. Recovery took place. In the second case one very small bleeding point was found on the posterior wall of the stomach near the pylorus. It could scarcely be called an ulcer, but as the edges were ecchymosed and the blood could be seen oozing from it, it was sutured as in the first case. Before operation the patient had lost a good deal of blood, and she was transfused as soon as the operation was over. She recovered.

The third case was a man, forty-two years of age, who had been suffering from pain after food, vomiting and loss of flesh, and latterly from hæmatemesis and melæna. Laparotomy was performed with the object of ascertaining whether there was a malignant growth. Nothing was found, and the abdomen was closed. On recovering from the anæsthetic, however, hæmatemesis came on and the next day the abdomen was reopened, the stomach was drawn into the wound, packed round with sponges, and opened as in the other cases. For a long time careful search failed to reveal the source of the bleeding, but at last a large irregular surface, upwards of an inch and a half in length, was found at the cardiac end, from which blood was pouring freely. On attempting to ligature the base of this, as in the other cases, it was found that the silk cut through at once, and only made the bleeding worse; therefore, there being no adhesions on the serous side, it was determined to ligature the whole thickness of the stomach wall. The ulcerated portion was inverted into the interior of the stomach and a silk ligature was tied as tightly as possible round the base of the projecting cone. This controlled the bleeding at once, and then the puckered peritoneal surface was brought together with a Lembert's suture. All went well for fifteen days, when the patient was sick and brought up a good deal of blood. After this, however, there was no recurrence; there was no more pain or vomiting, and he left the hospital perfectly well.

Commenting on these cases Mr. Mansell Moullin said that it was not unreasonable to think that if this operation could be shown to be unattended by any great amount of risk, there might be many people who would prefer the chance of an immediate and perfect cure to months, and even years, of comparative starvation, with the possibility of sudden death always before them, and at best a doubtful hope that recovery would ever be perfect. Not that he suggested operation in cases in which the amount of blood lost was not sufficient to be serious, or in which there had been only a single attack. But when the bleeding returned more than once and was either so profuse as to place life in immediate peril, or so continuous as to threaten dangerous anæmia, operation with a view not merely of stopping bleeding once for all, but of getting rid of the ulcer, seemed to offer a better prospect than persistence in other forms of treatment.

The discussion on this paper was postponed to a future date.

